# Meta-Analysis of the Correlation between Apparent Diffusion Coefficient and Standardized Uptake Value in Malignant Disease

**DOI:** 10.1155/2017/4729547

**Published:** 2017-02-26

**Authors:** Shengming Deng, Zhifang Wu, Yiwei Wu, Wei Zhang, Jihui Li, Na Dai, Bin Zhang, Jianhua Yan

**Affiliations:** ^1^Department of Nuclear Medicine, The First Affiliated Hospital of Soochow University, Suzhou, China; ^2^Department of Nuclear Medicine, First Hospital of Shanxi Medical University, Taiyuan, China; ^3^Molecular Imaging Precision Medicine Collaborative Innovation Center, Shanxi Medical University, Taiyuan, China

## Abstract

The objective of this meta-analysis is to explore the correlation between the apparent diffusion coefficient (ADC) on diffusion-weighted MR and the standard uptake value (SUV) of ^18^F-FDG on PET/CT in patients with cancer. Databases such as PubMed (MEDLINE included), EMBASE, and Cochrane Database of Systematic Review were searched for relevant original articles that explored the correlation between SUV and ADC in English. After applying Fisher's *r*-to-*z* transformation, correlation coefficient (*r*) values were extracted from each study and 95% confidence intervals (CIs) were calculated. Sensitivity and subgroup analyses based on tumor type were performed to investigate the potential heterogeneity. Forty-nine studies were eligible for the meta-analysis, comprising 1927 patients. Pooled *r* for all studies was −0.35 (95% CI: −0.42–0.28) and exhibited a notable heterogeneity (*I*^2^ = 78.4%; *P* < 0.01). In terms of the cancer type subgroup analysis, combined correlation coefficients of ADC/SUV range from −0.12 (lymphoma, *n* = 5) to −0.59 (pancreatic cancer, *n* = 2). We concluded that there is an average negative correlation between ADC and SUV in patients with cancer. Higher correlations were found in the brain tumor, cervix carcinoma, and pancreas cancer. However, a larger, prospective study is warranted to validate these findings in different cancer types.

## 1. Introduction

At present, various imaging modalities play an important role in diagnosis, staging, follow-up, and therapeutic evaluation of patients with cancer. Positron emission tomography/computed tomography with F-18 based fluorodeoxyglucose (^18^F-FDG PET/CT) is considered as an accurate method for characterizing tumor lesions due to the availability of anatomic and glucose metabolic information of tumor [1]. The standardized uptake value (SUV) is the most frequently used parameter derived from ^18^F-FDG PET, which has been used for assessing tumor aggressiveness, differentiating benign from malignant tumors, and monitoring treatment [2, 3].

Magnetic resonance imaging (MRI) is another important tool to detect and characterize tumors. Specifically, diffusion-weighted imaging (DWI) provides an additional promising dimension to the conventional anatomical MRI. Apparent diffusion coefficient (ADC) is a parameter obtained by MR-DWI, reflecting the Brownian movement of water molecules. The ADC value has been shown to link with the cell density, microvascular circulation, and membrane integrity of a tumor tissue [4].

Although glucose metabolism and cell density represent two different facets of tumor biology, many researchers tried to find the relationship between ADC and SUV. However, there is a controversy in this relationship. Some data demonstrated that there was no significant correlation observed between SUV and ADC [5], while other studies reported that SUV was inversely correlated with ADC [6, 7]. Given the conflicting evidence on this issue, we conducted this meta-analysis to explore the correlation between ADC and SUV in cancer patients.

## 2. Methods

### 2.1. Literature Search

Two observers independently searched the PubMed (MEDLINE included), EMBASE, and Cochrane Library databases for published studies. The search was limited to publications written in English. The databases were searched using the terms ((positron emission tomography) OR (PET) OR (positron emission tomography/computed tomography) OR (PET/CT) OR (PET-CT) OR (positron emission tomography-computed tomography)) AND ((18F-FDG) OR (fluorodeoxyglucose) OR (FDG) OR (18FDG) OR (FDG-F18)) AND ((apparent diffusion coefficient) OR (ADC)) AND ((Diffusion Magnetic Resonance Imaging) OR (Diffusion MRI) OR (Diffusion Weighted MRI) OR (DWI) OR (diffusion-weighted magnetic resonance imaging) OR (MRI DWI) OR (diffusion-weighted imaging) OR (diffusion-weighted MRI)).

### 2.2. Study Identification and Selection

Two independent reviewers evaluated the potentially relevant articles on the basis of the inclusion and exclusion criteria. Articles were included if they met the following criteria:

- Investigation of the relationship between ADC measured by MR and SUV measured with PET or PET/CT scanning
- Studies focusing on patients with malignant tumors, which may include patients with benign conditions as long as the vast majority of patients (>50%) in the study had cancer
- Research article published in the peer-reviewed journals

The exclusion criteria included the following:

- Data or part of data presented in more than one article (in this case, the article containing the latest and/or the most complete data was chosen)
- Animal studies, reviews, case report, letters, editorials, abstracts, comments, and in vitro studies
- Studies including less than 10 patients or 10 lesions
- Articles without sufficient information for calculation of correlation coefficient

If there was discordance among the 2 independent researchers for one study, its eligibility was decided by the 3rd investigator.

### 2.3. Data Extraction

The data were extracted from the included literatures by two investigators (Shengming Deng and Bin Zhang) independently, and the extracted contents included the following:

- Overall characteristics of studies, including authors, year of publication, number of patients and lesions, and tumor type
- Technical characteristics of PET or PET/CT measurement of ^18^F-FDG, including characteristics of the scanner, ^18^F-FDG dose, uptake time of the tracer, emission scan time, delineation of the tumor, and indexes of uptake (SUV_max_, SUV_mean_, or others)
- Technical characteristics of MR or PET/MR measurement ADC covered imaging equipment, *b* value, MRI field strength, and the index used to characterize the ADC (average, minimum, or others)
- The degree of correlation between ADC and SUV, including Spearman's correlation coefficient (SCC), Pearson's correlation coefficient (PCS), and* r*^2^. If the article did not report the value of correlation coefficient *r* directly, *r* value was calculated based on the raw data or scatter plot using the free software Engauge Digitizer (free software downloaded from https://sourceforge.net) and the SPSS 18.0 software. SCC was used for this meta-analysis. Since the SCC has already been processed by logarithmic conversion, it does not need to undergo the conversion again. The published PCSs were converted to SCCs for further analysis [57]. The sampling of SCC is not normally distributed. Because its confidence interval (CI) depends on the value of correlation coefficient, we converted the SCC by Fisher transformation to obtain *z* value with an approximately normal distribution. *z* value was then converted by inverse Fisher transformation to obtain the SCC and the corresponding CI.

If more than one correlation coefficient value calculated according to various SUV indexes or ADC indexes was reported in the article, the lowest value was chosen.

When disagreements occurred between the two reviewers, a third investigator joined to vote for a decision.

### 2.4. Methodology of Quality Assessment

Two investigators (Shengming Deng and Bin Zhang) assessed the quality of the articles independently according to the QUADAS-2 [58], which consists of 2 parts of contents: “risk assessment” and “practical application.” The former was assessed from 4 key domains as patient selection, index test, reference standard, and flow and timing, and the latter included 3 aspects as patient selection, index test, and reference standard.

To make sure that the QUADAS-2 tool is applicable to the present study, we designated SUV measurement as the “reference test” and ADC measurement as the “index test.” In this study, we chose one month as the threshold interval between PET or PET/CT examination and DWI-MRI detection in case tumor biology will change much. A third reviewer was introduced when there were assessing differences between the two observers.

### 2.5. Meta-Analysis

The pooled correlation coefficient between SUV and ADC was calculated according to the values of correlation coefficients obtained in each individual study. Correlation coefficient values were converted by Fisher's *r*-to-*z* transformation to obtain approximately normally distributed *z* values to further calculate 95% CIs. The random-effects model was used for the pooled analysis in this study. Correlations were classified as poor (correlation coefficient *r* < 0.20), average (*r* = 0.20–0.39), moderate (*r* = 0.40–0.59), significant (*r* = 0.60–0.79), and strong (*r* > 0.80) [59]. Publication bias was assessed by means of Begg's funnel plots and Begg's statistical test.

The heterogeneity of *r* values between studies was tested by calculating *Q* statistic and the inconsistency index (*I*^2^). *p* < 0.05 or* I*^2^ > 50% indicated the presence of heterogeneity. In case of the existence of heterogeneity, a sensitivity analysis was performed for all studies to further investigate the study heterogeneity. In a subgroup analysis, studies were stratified according to tumor type and correlation coefficient value (SUV_mean_/ADC_mean_, SUV_max_/ADC_min_, SUV_max_/ADC_mean_, etc.).

Statistical analysis was performed using STATA 11 software package (Stata Corporation, College Station, TX, USA). *p* < 0.05 was considered statistically significant.

## 3. Results

### 3.1. Literature Search and Selection of Studies

**Figure 1:**
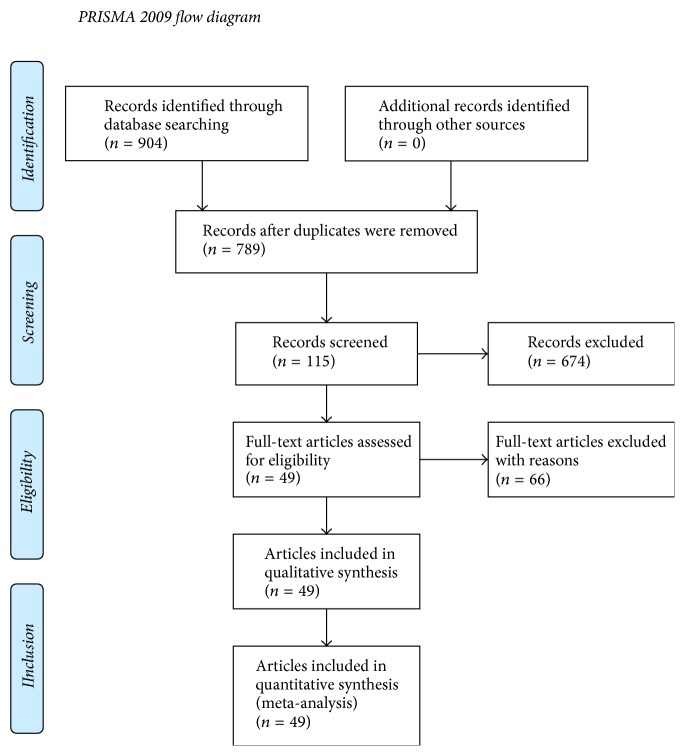
Flow diagram of study selection.

The original search identified 145 articles in PubMed and 759 articles in EMBASE. After removing duplicates, 789 abstracts were screened according to the evaluation criteria, and 115 in total were selected to be read in full as potentially eligible. After reading the full texts, 66 studies were excluded for the following reasons: (1) the article did not involve the evaluation of the relationship between ADC value and ^18^F-FDG uptake (*n* = 38); (2) the number of cases or tumor sites studied was fewer than 10 (*n* = 13); (3) the raw data in the article failed to generate the correlation coefficient values (*n* = 10); (4) part of the data in the study appeared in other articles (*n* = 3); (5) parameters measured by two individual reviewers were presented in the article which was difficult to choose (*n* = 1); and (6) most of the cases studied were benign tumors (*n* = 1).  Figure 1 describes the study selection process and results according to the PRISMA guidelines. Finally, 49 published articles were included in the present study [8–56].

### 3.2. Study Characteristics

The selected studies were published between 2008 and 2015. The median number of patients per study was 32 (range: 7–131) with a total number of 1927 patients. In some studies, more than one tumor site was analyzed on several patients; therefore, a total of 2356 samples were assessed in the meta-analysis. Studies covering a range of cancer sites are summarized in Table 1.

The most studied tumor location was the lung with 10 studies. The second tumor type was head and neck cancer, for which there were 6 studies. Five groups studied breast cancer, lymphoma, and cervical cancer. Other tumor types include metastatic gastrointestinal stromal tumors (GIST), brain cancer, hepatocellular cancer, esophageal cancer, peritoneal carcinomatosis, pancreatic cancer, and gastric, rectal, uterus, hepatocellular, and various types.

For MR-DWI examination, forty-one studies used a stand-alone MR scanner, while 8 studies used a PET/MR scanner. For MRI field strength, twenty-four studies used 1.5 T, twenty-three studies used 3.0 T, and 2 studies used both. For the index of ADC, twenty-six studies used ADC_mean_, fifteen studies used ADC_min_, and 8 studies used other indexes. For ^18^F-FDG PET scan, SUV_max_, SUV_mean_, and other SUV were used to calculate *r* values in 29, 11, and 9 studies, respectively (Table 2).

### 3.3. The Results of QUADAS-2 Assessing the Quality of the Included Articles

**Figure 2:**
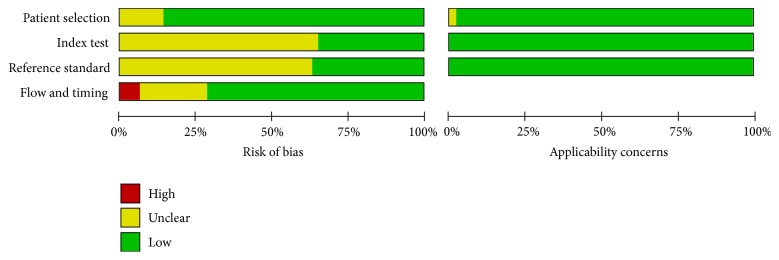
Methodological quality of all eligible studies.

As shown in Figure 2, the results of QUADAS-2 assessing the quality of the included articles indicated that the results of 10 studies adequately addressed all risk of bias domains. Among all the 49 studies, risk of bias was high or unclear with regard to patient selection for 7 studies, the index text for 32 studies, the reference standard for 31 studies, and flow and timing for 14 studies.

Interpretation of ADC or SUV in a blinded fashion was an item which most studies did not report. Seventeen studies clearly stated that the index test was assessed without knowledge of the results of the reference standard, while this was unclear in 32 studies. Similarly, in 18 studies, the interpretation of reference standard was clearly stated as under unknown index test, while the other 31 studies did not state the interpretation of reference standard clearly.

Acceptable delay between reference and index tests was the item which many studies did not report. Eleven studies provided no information about the time interval between the execution of MR-DWI and the ^18^F-FDG PET/CT scan. In 3 studies documented, the interval was more than 4 weeks.

In addition, patients enrolled in 1 study were investigated on residual tumors after completion of therapy. In these patients, whether the relationship between ^18^F-FDG uptake and ADC value differs from that in patients with pretherapeutic tumor is unclear; therefore, the risk of case selection bias in this study was considered unclear in the present analysis.

### 3.4. The Results of a Meta-Analysis

The data provided by the finally chosen studies all met the standard of meta-analysis. *r* values for 3 studies were calculated from provided *r*^2^, and *r* values for 2 other studies were determined from the provided scatter plot. For 3 other studies, *r* values were calculated based on the provided raw data of corresponding ADC and SUV.

**Figure 3:**
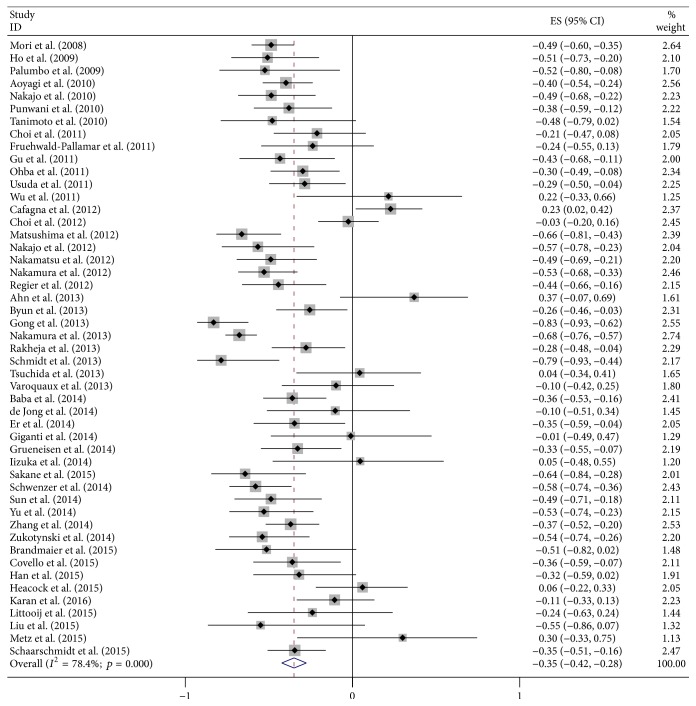
Forest plots of the summary correlation coefficient (*r*) with corresponding 95% CIs for the correlation between SUV and ADC in all eligible studies.

**Figure 4:**
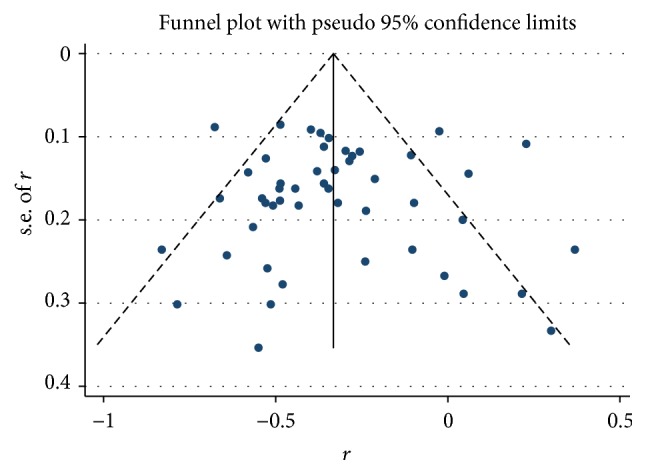
The funnel plot of the publication bias.

Final combined *r* value calculated from all the included articles was −0.35 (95% CI: −0.42–−0.28), but the results of heterogeneity test indicated the presence of marked heterogeneity among studies (*I*^2^ = 78.4%; *p* < 0.01; Figure 3). We then conducted a sensitivity analysis by excluding each article at a time to observe its effect on the final outcome, but the results showed that no individual study contributed more greatly to the total heterogeneity. The results of Begg's test indicated no significant publication deviation among the included articles (*p* > 0.05; Figure 4).

**Figure 5:**
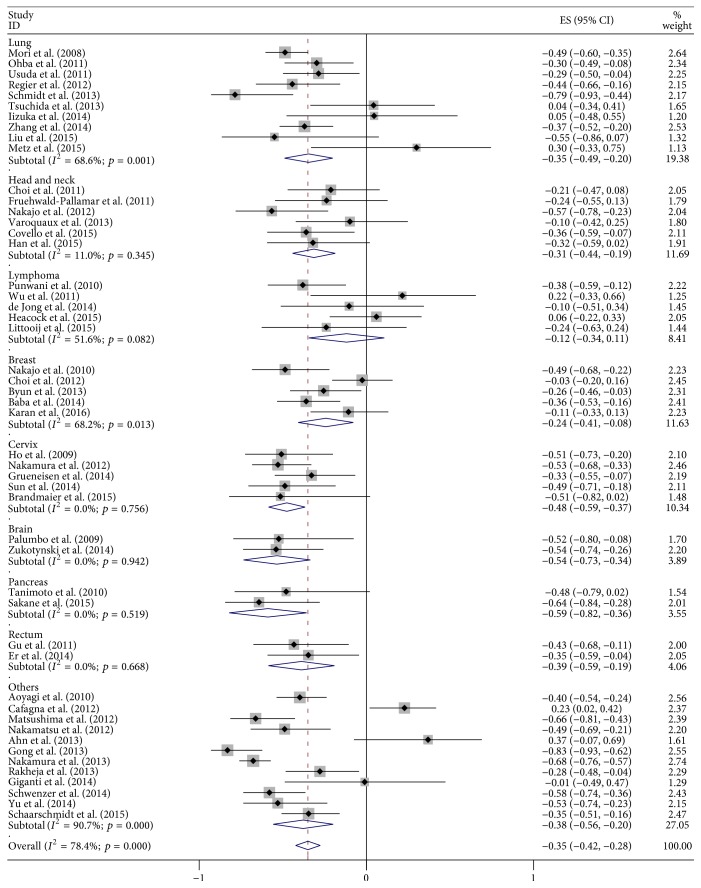
Forest plot of subgroup analysis based on cancer type.

As shown in Figure 5, the subgroup analysis for tumor types showed that combined *r* for the 10 studies of lung cancer was −0.35 (95% CI: −0.49–−0.20), and there was significant heterogeneity among the included studies (*I*^2^ = 68.6%; *p* < 0.01). Combined *r* value for the 6 studies on head and neck cancer was −0.31 (95% CI: −0.44–−0.19;* I*^2^ = 11.0%; *p* > 0.05) which displayed no heterogeneity. Combined *r* value for the subgroup of 5 studies on lymphoma and cervical cancer was −0.12 (95% CI: −0.34–0.11) and −0.48 (95% CI: −0.59–−0.37), respectively, without significant heterogeneity ((*I*^2^ = 51.6%; *p* > 0.05) and (*I*^2^ = 0.0%; *p* > 0.05)). Combined *r* value for the 5 studies on breast cancer was −0.24 (95% CI: −0.41–−0.08;* I*^2^ = 68.2%; *p* < 0.01).

**Figure 6:**
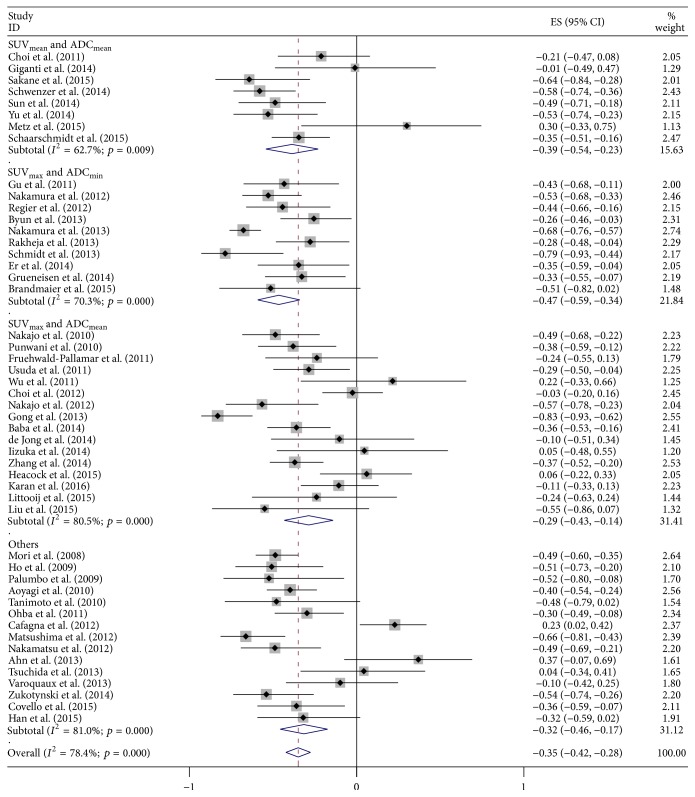
Forest plot of subgroup analysis based on correlation coefficient value.

Results for the subgroup analysis based on correlation coefficient value are shown in Figure 6. Eight studies in SUV_mean_/ADC_mean_ resulted in *r* = −0.39 (95% CI: −0.54–−0.23), with* I*^2^ = 62.7% (*p* < 0.01). Pooled *r* for ten studies in SUV_max_/ADC_min_ was −0.47 (95% CI: −0.59–−0.34), with* I*^2^ = 70.3% (*p* < 0.01). In SUV_max_/ADC_mean_, sixteen studies provided *r* = −0.29 (95% CI: −0.43–−0.14) with* I*^2^ = 80.5% (*p* < 0.01).

## 4. Discussion

In the recent years, the correlation between ADC and SUV has been increasingly studied. In the present study, we investigated the relationship between ^18^F-FDG uptake and ADC value using meta-analysis methods. Our meta-analysis showed that, in cancer patients, there was an average negative correlation between the SUV and ADC. Subgroup analysis on different tumor types indicated that degrees of correlation among different tumor types varied and heterogeneity of some subgroups changed significantly. The subgroup analysis on various correlation coefficient values indicated that combined *r* values of subgroups did not show significant changes, and there were no significant changes in heterogeneity.

In this study, we used QUADAS-2 as an evidence-based quality assessment tool. In the present analysis, the vast majority of the articles did not mention whether the test results of DWI-MRI and ^18^F-FDG PET (or PET/CT) are interpreted blindly. In most studies, the time interval between ^18^F-FDG PET (or PET/CT) imaging and the acquirement of ADC was not clearly stated. In addition, some studies did not address the inclusion criteria of patients adequately. The above problems may increase the bias of study.

DWI provides an excellent tissue contrast through detection of differences in the Brownian motion of water molecules in tissues. ADC is a parameter calculated from DWI and altered by any architectural changes in the proportion of extracellular to intracellular water molecules because the diffusion of water molecules is disturbed by intracellular organelles and macromolecules [60]. Malignant tumors usually show decreased ADC values because they are characterized by increased cellularity, larger nuclear/cytoplasmic ratio, and less extracellular space relative to normal tissues which restrict the diffusion of water molecules [61]. Currently, ^18^F-FDG PET/CT has been considered as the standard of care in various cancers. ^18^F-FDG uptake is correlated with the number of viable tumor cells and their metabolic activity. Glucose utilization in tumors is increased due to the Warburg effect [62]. Recently, the introduction of simultaneous PET/MRI makes it possible to combine functional and metabolic studies in malignancies in one examination. It was postulated that there is a correlation between ADC and SUV. The present study showed that the pooled correlation coefficient between SUV and ADC was −0.35, indicating an average negative correlation. A possible explanation of this result might be that although there is a certain overlap of the information provided by ^18^F-FDG PET and DW-MRI, the two parameters (SUV and ADC) reflect different tumor biology. For example, except for cellularity, ADC is correlated directly with tumor necrosis because of increased presence of free water in the necrotic area [63]. However, ^18^F-FDG PET demonstrates tumor necrosis as photopenic defects. In addition, although the ADC measurement is derived from DWI which is an MR sequence that is known for a high detection rate of lesions, it is not always very specific [64]. Our result suggested that ^18^F-FDG PET and DWI-MRI might complement each other on the clinical diagnosis.

We conducted a subgroup analysis based on different tumor types. The meta-analysis about ADC and tumor cellularity correlation revealed no notable variation between the subgroups based on cancer type [65]. In this study, our results showed that the correlation between ^18^F-FDG SUV and ADC differed between histological types. Combined correlation coefficients range from −0.12 (lymphoma, *n* = 5) to −0.59 (pancreatic cancer, *n* = 2). Correlation was moderate in brain, cervix, and pancreas, average in lung, head and neck, breast, and rectum, and poor in lymphoma. However, this issue needs to be further explored with more experiments.

The present study has some potential limitations. First, although the number of patients included in this study was large, they were relatively limited to a certain type of tumors. This may cause limitations in our inference based on the results of subgroup analysis on different histological types. Second, our meta-analysis was based only on published studies which provided *r* values or raw data which can be used to calculate *r* values. Other articles which only report positive or negative results without specific data were excluded from this analysis. In addition, this study was restricted to articles published in English, which would cause publication bias. However, the results of Begg's test showed no evidence of publication bias. We also used the random-effects model to reduce heterogeneity. Therefore, the results of the present study are reliable.

In short, although there are limitations in this study, our meta-analysis demonstrated an average negative correlation between the SUV and ADC values in patients with cancer. Sufficient data support a moderate correlation for brain, cervix, and pancreas, average correction for lung, head and neck, breast, and rectum, and poor for lymphoma. However, a prospective study with a larger population is warranted to validate these findings in different cancer types.

## Figures and Tables

**Table 1:** ^18^F-FDG PET scan characteristics and MRI scanner.

Author	Year	Scanner	FDG dose (MBq)	Uptake period (min)	Emission time (min)	SUV index	Delineation
Mori et al. [8]	2008	GE Discovery ST PET/CT + Philips Intera Achieva Nova Dual 1.5 T MR	3.7/kg	60	3	SUV-CR	Manual
Ho et al. [9]	2009	GE Discovery ST16 PET/CT + Siemens Tim Trio 3.0 T MR	333–407	50	3	SUV_max_/SUV_mean_	Automatic
Palumbo et al. [10]	2009	GE Advance PET + GE 1.5 T MR	444–555	45	6–10	SUV-CR	Semiautomatic
Aoyagi et al. [11]	2010	PET + Philips Intera Achieva Nova Dual 1.5 T MR	N	N	N	SUV_max_	N
Nakajo et al. [12]	2010	GE Discovery STE PET/CT + Philips Intera Achieva 1.5 T MR	3.7/kg	60	2.5	SUV_max_	Automatic
Punwani et al. [13]	2010	GE Discovery LS PET/CT + Siemens Avanto 1.5 T MR	370	60	N	SUV_max_	Manual
Tanimoto et al. [14]	2010	Toshiba Aquiduo PET/CT + GE Signa HDx 3.0 T MR	217.8–372.5	60	3	SUV	Automatic
Choi et al. [15]	2011	Philips Gemini or Siemens Biograph 40 PET/CT + GE Signa HDx or HDxt 1.5 T MR	5.2/kg	60	2	SUV_mean_	Manual
Fruehwald-Pallamar et al. [16]	2011	Siemens Biograph 64 PET/CT + Philips Achieva 3.0 T MR	300	50	3	SUV_max_	Automatic
Gu et al. [17]	2011	GE Discovery VCT PET/CT + Philips Achieva 3.0 T MR	4.8/kg	60	4	SUV_max_	Automatic
Ohba et al. [18]	2011	GE Discovery ST PET/CT + Philips Achieva 3.0 T or Philips Gyroscan Intera Achieva Nova Dual 1.5 T MR	3.7/kg	60	3	SUV-CR	N
Usuda et al. [19]	2011	Siemens Biography Sensation 16 PET/CT + Siemens Magnetom Avanto 1.5 T MR	185	60	3	SUV_max_	Automatic
Wu et al. [20]	2011	GE Discovery STE 16 PET/CT + Siemens Trio-Tim 3.0 T MR	370	60	3	SUV_max_	Manual
Cafagna et al. [21]	2012	GE Discovery STE 16 PET/CT + Philips Achieva 1.5 T MR	3.7/kg	60	3	SUV_max_	N
Choi et al. [22]	2012	Siemens Biograph Duo or Biograph Truepoint PET/CT + Philips Achieva 1.5 T or Siemens Magnetom Verio 3.0 T MR	N	N	2-3	SUV_max_	N
Matsushima et al. [23]	2012	Toshiba Aquiduo PCA-7000B PET/CT + GE Signa Excite HDxt 1.5 T MR	3.7/kg	60	6	SUV-CR	Manual
Nakajo et al. [24]	2012	GE Discovery STE PET/CT + Siemens Magnetom Avanto 1.5 T MR	3.7/kg	60	N	SUV_max_	Automatic
Nakamatsu et al. [25]	2012	Toshiba Aquiduo 16 PET/CT + Siemens Magnetom Symphony 1.5 T MR	166.7–320.8	60	2	SUV_mean_	Manual
Nakamura et al. [26]	2012	Siemens Biograph LS/Sensation 16 PET/CT + Siemens Magnetom Avanto 1.5 T MR	3.7/kg	90	2.4	SUV_max_	Manual
Regier et al. [27]	2012	Philips Gemini GXL 10 PET/CT + Philips Achieva 1.5 T MR	5/kg	60	1–1.5	SUV_max_	N
Ahn et al. [28]	2013	Siemens Biograph Truepoint 40 PET/CT + Siemens Magnetom Tim Trio 3.0 T MR	5.5/kg	45	N	SUV_max_	N
Byun et al. [29]	2013	Siemens Biograph 6 PET/CT + Siemens Magnetom TrioA Tim 3.0 T MR	7.4/kg	60	3.5	SUV_max_	Automatic or manual
Gong et al. [30]	2013	GE Discovery VCT PET/CT + Philips Achieva 3.0 T MR	4.8/kg	60	4	SUV_max_	Manual
Nakamura et al. [31]	2013	Siemens Biograph LS/Sensation 16 PET/CT + Siemens Magnetom Avanto 1.5 T MR	3.7/kg	90	2.4	SUV_max_	Manual
Rakheja et al. [32]	2013	Siemens Biograph mCT PET/CT + Siemens Biograph mMR PET/MR	555	45	2	SUV_max_	Manual
Schmidt et al. [33]	2013	Siemens HI-REZ Biograph 16 or Siemens Biograph mCT PET/CT + Siemens Biograph mMR PET/MR	317–381	55–61	2-3	SUV_max_	N
Tsuchida et al. [34]	2013	GE Discovery LS4 PET/CT + GE Signa Excite 1.5 T MR	185	50	2	SUV_mean_	N
Varoquaux et al. [35]	2013	Siemens Biograph 16-slice PET/CT + Siemens Espree 1.5 T or Trio 3.0 T MR	370	60	3	SUV	Manual
Baba et al. [36]	2014	GE Advance NXi PET/CT + Philips Intera Achieva 1.5 T MR	3.7/kg	60	2	SUV_max_	Manual
de Jong et al. [37]	2014	Siemens Biograph 40 True Point or Philips Gemini TOF PET/CT + Philips Achieva or Siemens Magnetom Avanto 1.5 T MR	2.0–3.7/kg	60–75	2-3	SUV_max_	Manual
Er et al. [38]	2014	GE Discovery ST PET/CT + Siemens Magnetom Verio 3.0 T MR	5.55/kg	50–60	N	SUV_max_	Manual
Giganti et al. [39]	2014	GE Discovery ST, Discovery STE, Discovery-690, or Philips Gemini GXL PET/CT + Philips Achieva 1.5 T MR	3.7/kg	60	2.5	PVC-SUV_mean_	Automatic
Grueneisen et al. [40]	2014	Siemens Biograph mMR PET/MR	201 ± 69	102 ± 39	8	SUV_max_	Manual
Iizuka et al. [41]	2014	GE Discovery ST Elite PET/CT + Siemens Avanto 1.5 T MR	3.7/kg	60	2-3	SUV_max_	N
Sakane et al. [42]	2015	Philips Gemini GXL PET/CT + GE Signa HDxt 3.0 T MR	3.7/kg	60	2	SUV_mean_	Manual
Schwenzer et al. [43]	2014	PET/CT + Siemens Biograph mMR PET/MR	294–386	62 ± 4	6	SUV_mean_	Manual
Sun et al. [44]	2014	Philips Ingenuity TF PET/MR	240–350	60 ± 12	4	SUV_mean_	Automatic
Yu et al. [45]	2014	GE Discovery VCT PET/CT + Philips Achieva 3.0 T MR	4.8/kg	60	2.5	SUV_mean_	Manual
Zhang et al. [46]	2014	Siemens Biograph 40 PET/CT + Siemens Trio-Tim 3.0 T MR	5.55/kg	60	N	SUV_max_	N
Zukotynski et al. [47]	2014	GE Advance NXi, Discovery LS, and Discovery STE; Philips G-PET; Siemens HR1 and HI-REZ Bioscan PET + 1.5 T MR	5.55/kg	40–60	10	SUV_mean_/WM	Manual
Brandmaier et al. [48]	2015	Siemens Biograph mMR 3.0 T PET/MR	309 ± 70.32	130	5	SUV_max_	Manual
Covello et al. [49]	2015	Philips Gemini TF PET/CT + Siemens Biograph mMR 3.0 T MR	406 ± 40	81 ± 15	3	SUV	Automatic
Han et al. [50]	2015	GE Discovery STE PET/CT + GE Signa HDxt 1.5 T MR	5/kg	60	2.5	SUV_mean_	Manual
Heacock et al. [51]	2015	Siemens Biograph mCT PET/CT + Siemens Biograph mMR 3.0 T PET/MR	506.9–566.1	45	2-3	SUV_max_	Manual
Karan et al. [52]	2016	GE Discovery STE 8 PET/CT + Siemens Avanto 1.5 T MR	296–370	60	2.5	SUV_max_	Automatic
Littooij et al. [53]	2015	Siemens Biograph 16 or Biograph 40 Truepoint, Philips Gemini TOF or Allegro PET-CT + Philips Achieva, Siemens Avanto or GE Signa 1.5 T MR	2–3.7/kg	60	N	SUV_max_	N
Liu et al. [54]	2015	Siemens Biograph 40 PET/CT + GE Signa HDE 1.5 T MR	5.55/kg	60	N	SUV_max_	N
Metz et al. [55]	2015	Siemens Biograph Sensation 16 PET/CT + Siemens Magnetom Avanto 1.5 T MR	456 ± 25	64 ± 3	2	SUV_mean_	Manual
Schaarschmidt et al. [56]	2015	Siemens mCT™ PET/CT + Siemens Biograph mMR PET/MR	280 ± 50	58 ± 11	2	SUV_mean_	Manual

N: not reported.

**Table 2:** MRI characteristics, cancer types, and *r* values.

Author	Year	Nation	Number of patients	Number of tumors	Tumor	Age	Design	Field	Index	*b* value (s/m^2^)	*r*
Mori et al. [8]	2008	Japan	104	140	Lung (various)	Adult	Prospective	1.5 T	ADC_min_	1000	−0.504
Ho et al. [9]	2009	Taiwan	33	33	Cervix (various)	Adult	Prospective	3.0 T	ADC_min_/ADC_mean_	0 and 1000	−0.526
Palumbo et al. [10]	2009	USA	15	18	Brain (metastases)	Adult	N	1.5 T	ADC-CR	N	−0.524
Aoyagi et al. [11]	2010	Japan	123	123	Esophageal (SCC)	Adult	N	1.5 T	ADC	0 and 1000	−0.398
Nakajo et al. [12]	2010	Japan	44	44	Breast (ductal carcinoma)	Adult	Retrospective	1.5 T	ADC_mean_	0 and 1000	−0.486
Punwani et al. [13]	2010	UK	16	53	Lymphoma (MCL)	Children	N	1.5 T	ADC_mean_	500	−0.38
Tanimoto et al. [14]	2010	Japan	16	16	Pancreas (various)	Adult	N	3.0 T	ADC	400, 800, and 1200	−0.48
Choi et al. [15]	2011	Korea	47	47	Head and neck (SCC)	Adult	Retrospective	1.5 T	ADC_mean_	1000	−0.222
Fruehwald-Pallamar et al. [16]	2011	Austria	31	31	Head and neck (SCC)	Adult	Prospective	3.0 T	ADC_mean_	0 and 800	−0.238
Gu et al. [17]	2011	China	33	33	Rectum (adenocarcinoma)	Adult	N	3.0 T	ADC_min_	0 and 1000	−0.45
Ohba et al. [18]	2011	Japan	58	76	Lung (various)	N	Prospective	1.5 T	ADC_min_	1000	−0.31
Usuda et al. [19]	2011	Japan	63	63	Lung (various)	Adult	N	1.5 T	ADC_mean_	0 and 800	−0.286
Wu et al. [20]	2011	Finland	15	15	Lymphoma (DLBCL)	Adult	Prospective	3.0 T	ADC_mean_	0 and 800	0.215
Cafagna et al. [21]	2012	Italy	38	88	Various	N	Retrospective	1.5 T	ADC	500 and 1000	0.238
Choi et al. [22]	2012	Korea	118	118	Breast (IDC)	Adult	N	1.5 and 3.0 T	ADC_mean_	0, 750, and 1000	−0.025
Matsushima et al. [23]	2012	Japan	36	36	Glioma and lymphoma	Children and adult	Retrospective	1.5 T	ADC_min_	1000	−0.68
Nakajo et al. [24]	2012	Japan	26	26	Head and neck (SCC)	Adult	Retrospective	1.5 T	ADC_mean_	0 and 800	−0.566
Nakamatsu et al. [25]	2012	Japan	24	41	Metastatic neck lymph nodes of head and neck (SCC)	Adult	Retrospective	1.5 T	ADC_min_	0 and 1000	−0.489
Nakamura et al. [26]	2012	Japan	66	66	Cervix (various)	Adult	N	1.5 T	ADC_min_	0, 50, and 1000	−0.529
Regier et al. [27]	2012	Germany	41	41	Lung (NSCLC)	N	Prospective	1.5 T	ADC_min_	0 and 500	−0.46
Ahn et al. [28]	2013	Korea	21	21	Liver (HCC)	Adult	Retrospective	3.0 T	ADC_max_	50, 400, and 800	0.369
Byun et al. [29]	2013	Korea	75	75	Breast (IDC)	Adult	Retrospective	3.0 T	ADC_min_	0 and 800	−0.267
Gong et al. [30]	2013	China	7	21	Metastatic GIST	Adult	Retrospective	3.0 T	ADC_mean_	0, 150, and 1000	−0.843
Nakamura et al. [31]	2013	Japan	131	131	Endometria	Adult	Prospective	1.5 T	ADC_min_	0, 50, and 1000	−0.677
Rakheja et al. [32]	2013	USA	24	69	Various	Adult	N	3.0 T	ADC_min_	0, 350, and 750	−0.29
Schmidt et al. [33]	2013	Germany	14	14	Lung (various)	Adult	N	3.0 T	ADC_min_	0 and 800	−0.8
Tsuchida et al. [34]	2013	Japan	28	28	Lung (various)	Adult	N	1.5 T	ADC	0 and 800	0.043
Varoquaux et al. [35]	2013	Switzerland	33	34	Head and neck (SCC)	Children and adult	Retrospective	1.5 and 3.0 T	ADC	0 and 1000	−0.103
Baba et al. [36]	2014	Japan	79	83	Breast (various)	Adult	Retrospective	1.5 T	ADC_mean_	1000	−0.36
de Jong et al. [37]	2014	Netherlands	21	21	Lymphoma (DLBCL)	Adult	Prospective	1.5 T	ADC_mean_	0 and 1000	−0.103
Er et al. [38]	2014	Turkey	41	41	Rectum (adenocarcinoma)	Adult	Retrospective	3.0 T	ADC_min_	50, 400, and 1000	−0.347
Giganti et al. [39]	2014	Italy	17	17	Gastric (adenocarcinoma)	Adult	Prospective	1.5 T	ADC_mean_	0 and 600	−0.01
Grueneisen et al. [40]	2014	Germany	15	54	Cervix (various)	Adult	Prospective	3.0 T	ADC_min_	0, 500, and 1000	−0.342
Iizuka et al. [41]	2014	Japan	15	15	Lung (NSCLC)	Adult	N	1.5 T	ADC_mean_	0, 500, and 1000	0.046
Sakane et al. [42]	2015	Japan	20	20	Pancreas (adenocarcinoma)	Adult	Retrospective	3.0 T	ADC_mean_	0 and 800	−0.66
Schwenzer et al. [43]	2014	Germany	20	52	Peritoneal carcinomatosis	Adult	Prospective	3.0 T	ADC_mean_	50 and 800	−0.58
Sun et al. [44]	2014	China	35	35	Cervix (various)	Adult	Prospective	3.0 T	ADC_mean_	0, 200, 500, and 1000	−0.505
Yu et al. [45]	2014	China	8	34	Peritoneal metastases	Adult	Prospective	3.0 T	ADC_mean_	0, 400, and 800	−0.548
Zhang et al. [46]	2014	China	113	113	Lung (various)	Adult	N	3.0 T	ADC_mean_	1000	−0.37
Zukotynski et al. [47]	2014	USA	36	36	Brain (BSG)	Children	Retrospective	1.5 T	ADC_mean_	5 and 1000	−0.54
Brandmaier et al. [48]	2015	Germany	14	14	Cervix (various)	Adult	Prospective	3.0 T	ADC_min_	0 and 800	−0.532
Covello et al. [49]	2015	Italy	44	44	Head and neck (various)	Adult	N	3.0 T	ADC_mean_	0, 500, and 800	−0.36
Han et al. [50]	2015	Korea	34	34	Head and neck (SCC)	Adult	Retrospective	1.5 T	ADC_min_	0 and 1000	−0.333
Heacock et al. [51]	2015	USA	13	51	Lymphoma (various)	Adult	Prospective	3.0 T	ADC_mean_	0, 350, and 750	0.06
Karan et al. [52]	2016	Turkey	70	70	Breast (IDC)	Adult	Retrospective	1.5 T	ADC_mean_	0, 200, 600, and 800	−0.112
Littooij et al. [53]	2015	Netherlands	11	19	Lymphoma (various)	Children and adult	Prospective	1.5 T	ADC_mean_	0 and 1000	−0.24
Liu et al. [54]	2015	China	11	11	Lung (various)	Adult	N	1.5 T	ADC_mean_	1000	−0.55
Metz et al. [55]	2015	Germany	12	12	Lung (metastatic NSCLC)	Adult	Prospective	1.5 T	ADC_mean_	50, 300, and 600	0.3
Schaarschmidt et al. [56]	2015	Germany	25	100	Lymph node metastases of NSCLC	Adult	Retrospective	3.0 T	ADC_mean_	0, 500, and 1000	−0.36

SCC: squamous cell carcinoma; NSCLC: non-small cell lung cancer; MCL: mantle cell lymphoma; DLBCL: diffuse large B cell lymphoma; HCC: hepatocellular carcinoma; BSG: brain stem glioma; IDC: invasive ductal carcinoma; N: no report.
